# A pilot study: virtual reality-based intervention to boost optimism and alleviate stress, anxiety, and depression in undergraduates

**DOI:** 10.3389/fpsyg.2026.1778010

**Published:** 2026-02-25

**Authors:** Yun-Ju Lai, Jiraporn Sangpara, Suppakorn Wankrathok, Ching Hwa Chang, Shun-Chun Yu, Amee Patel, Hung-Hsin Chen, Yuan Zhang, Jiabin Shen, Yan Wang

**Affiliations:** 1 School of Nursing, Zuckerberg College of Health Sciences, University of Massachusetts Lowell, Lowell, MA, United States; 2 Institute of Biomedical Sciences, Academia Sinica, Taipei, Taiwan; 3 Department of Psychology, College of Fine Arts, Humanities & Social Sciences, University of Massachusetts Lowell, Lowell, MA, United States

**Keywords:** anxiety, depression, optimism, stress, VR intervention

## Abstract

**Objective:**

This pilot randomized controlled trial evaluated the short- and long-term effectiveness of a Positive Cognitive Behavioral Therapy (PCBT)–based virtual reality (VR) intervention in enhancing optimism and improving mental health outcomes among undergraduate students with low baseline optimism, compared with a task-based VR control.

**Methods:**

Twenty-eight full-time undergraduate students with low optimism [Life Orientation Test–Revised (LOT-R) ≤ 13] were randomly assigned to a PCBT-based VR intervention (*n* = 15) or a task-based VR control (*n* = 13). Participants completed six weekly 30-min VR sessions. Optimism, perceived stress, anxiety, and depressive symptoms were assessed at baseline, post-intervention, and at 3- and 6-month follow-ups. Linear mixed-effects models adjusted for age, gender, and academic period were used to evaluate intervention effects across time.

**Results:**

All participants completed post-intervention assessments, and 25 (89.29%) completed the 6-month follow-up. The PCBT-based VR group demonstrated a significant short-term increase in optimism (*β* = 2.02, *p* = 0.01) and a significant reduction in depressive symptoms (*β* = −3.04, *p* = 0.01) compared with the control group. Anxiety showed a trend toward short-term reduction, while stress did not change significantly. Across the 6-month follow-up, the intervention group maintained higher overall optimism than controls; however, the group-by-time interaction was not significant, indicating similar long-term trajectories for optimism, stress, anxiety, and depression across groups.

**Conclusion:**

A PCBT-based VR intervention produced meaningful short-term improvements in optimism and depressive symptoms among undergraduates with low optimism, with optimism gains sustained for 6 months. These findings suggest that VR-delivered positive psychology interventions may be a feasible and engaging approach to support student mental health. Larger, multi-site trials are needed to confirm efficacy and optimize long-term effects.

## 1 Introduction

The COVID-19 pandemic, along with social distancing, prolonged isolation, and lockdown policies, has intensified a wide range of psychological issues, including stress, anxiety, fear, loneliness, insomnia, and depression (Ganesan et al., 2021). Students have been particularly vulnerable, as disruptions to both social and academic routines have contributed to heightened mental health challenges. Recent studies report elevated levels of anxiety, stress, and depression in this population, with approximately 40–50% of students experiencing clinically relevant symptoms (Gupta et al., 2021; Cao et al., 2020). National survey data showed that 77.6% of the United States (U.S.) undergraduates reported moderate to high stress levels during this period (American College Health Association, 2024). Primary stressors included academic pressure, financial concerns, relationship and family difficulties, and sleep problems (American College Health Association, 2024).

Stress is strongly associated with anxiety and depressive symptoms, and pandemic-related burdens may further intensify these risks (Valikhani et al., 2020). Chronic stress is also associated with unhealthy eating patterns and maladaptive coping strategies, which together increase vulnerability to depression (Liu et al., 2017; Evans et al., 2015). During the pandemic, approximately 40% of U.S. adults reported symptoms of anxiety or depression—three times the pre-pandemic rate (Ettman et al., 2020). These trends underscore the heightened mental health burden faced by undergraduate students and highlight the need to identify protective psychological factors and develop targeted interventions.

One such protective factor is optimism, defined as the expectation of positive future outcomes (Scheier et al., 1994), and widely regarded as both a personality trait and a core construct in positive psychology (Scheier et al., 1994; Conversano et al., 2010). Optimism is consistently linked to better mental and physical health, including greater stress resilience and faster recovery following major medical procedures such as cardiac bypass surgery (Ronaldson et al., 2014; Tindle et al., 2012). Among college students, optimism is negatively associated with anxiety, which in turn is linked to poorer academic performance (Singh and Jha, 2013). Our previous study also showed that optimism is inversely associated with stress, anxiety, and depression (Lai et al., 2024). Collectively, these findings highlight the potential value of interventions to enhance optimism and reduce mental health risks among student populations.

Emerging technologies such as virtual reality (VR) offer promising tools for promoting emotional regulation and psychological resilience (Li Pira et al., 2023). VR immerses users in controlled therapeutic environments designed to influence cognitive and emotional processes. Nature-based VR experiences, in particular, have demonstrated rapid psychological and physiological benefits, including reduced stress and anxiety among young adults and individuals with mental health disorders (O’Meara et al., 2020; Villani et al., 2007; Xu et al., 2024; Freeman et al., 2017; Felnhofer et al., 2019). These advantages make VR especially applicable for supporting undergraduates experiencing elevated stress and anxiety.

Integrating VR with Positive Psychology Interventions (PPIs) creates a novel therapeutic approach that leverages immersive environments to cultivate positive emotions, behaviors, and cognitions. PPIs involve structured, evidence-based practices that promote positive emotions and cognitions, and they have been shown to improve wellbeing and reduce depressive symptoms (Duan et al., 2022; Bolier et al., 2013; Sin and Lyubomirsky, 2009; Geschwind et al., 2019). A meta-analysis indicated that PPIs are more effective than other active psychological treatments in improving wellbeing among adults with depression (Lim and Tierney, 2023). When combined with VR delivery, such as through Positive Cognitive Behavioral Therapy (PCBT), these interventions have shown enhanced engagement and promising mental health benefits (Gao et al., 2022; Jeppesen et al., 2022).

**Figure 1:**
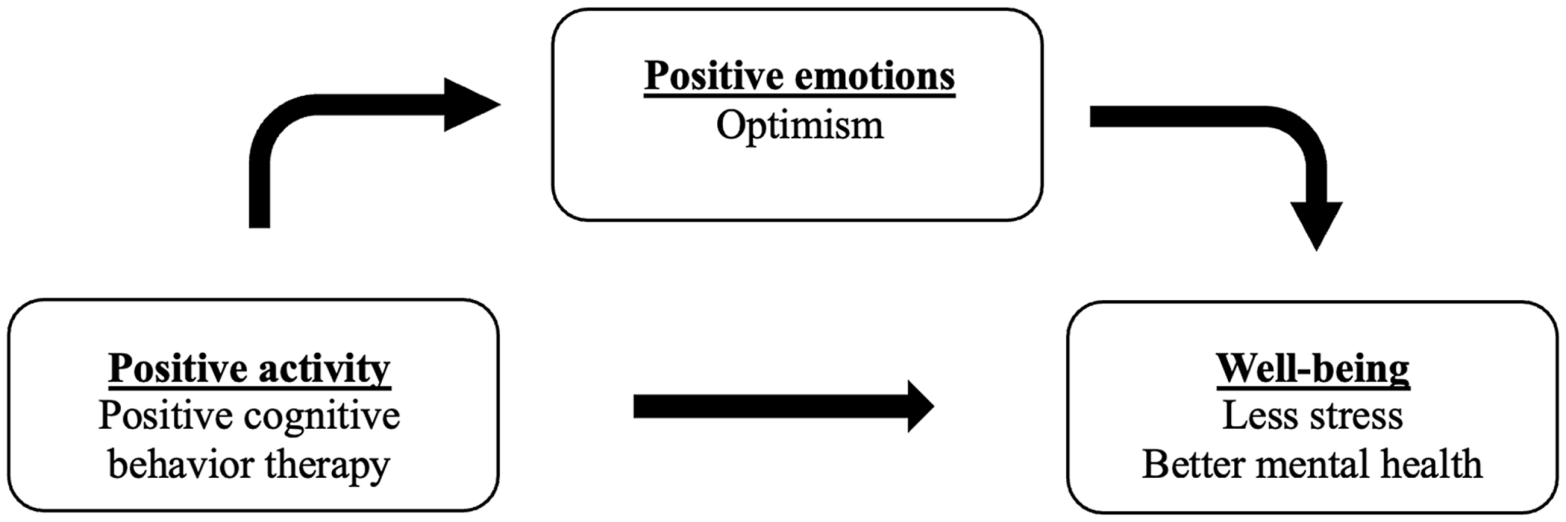
Conceptual framework adapted from Lyubomirsky and Layous’s positive-activity model (Lyubomirsky and Layous, 2013). The model illustrates how engaging in positive activities, such as VR-delivered PCBT exercises, promotes positive emotions and cognitions (e.g., optimism), which, in turn, enhance wellbeing outcomes, including reduced stress, anxiety, and depression.

This pilot study adapted Lyubomirsky and Layous’s Positive Activity Model (2013) as its conceptual framework to evaluate the efficacy of VR-delivered PPIs (Lyubomirsky and Layous, 2013). As illustrated in Figure 1, the model posits three interconnected components: (1) positive activities, here operationalized as PCBT-based VR interventions; (2) positive emotions, including heightened optimism; and (3) wellbeing outcomes, such as reduced stress and improved mental health. Our VR intervention applied PCBT-based activities within this framework to promote emotional and psychological benefits (Auyeung and Mo, 2019; Oh et al., 2019).

Despite growing evidence supporting VR and PPIs independently, the integration of VR-delivered PCBT to enhance optimism and mental health in college students remains underexplored. Therefore, this study examined the effectiveness of a VR-based PCBT intervention in increasing optimism and reducing stress, anxiety, and depression. We hypothesized that participants receiving the PCBT-based VR intervention would demonstrate significantly greater improvements in optimism and reductions in stress, anxiety, and depressive symptoms compared with those in the task-based VR control condition.

## 2 Materials and methods

### 2.1 Study design and setting

A two-arm randomized controlled trial (RCT) was conducted among undergraduate students at a public university in the northeastern U.S. between February 2024 and October 2025. Participants were recruited using multiple strategies while maintaining privacy and confidentiality. Study information was disseminated through university-wide and classroom announcements, and recruitment flyers were posted on on-campus bulletin boards accessible to students. Interested students contacted the research team and completed an initial screening to assess eligibility prior to enrollment.

### 2.2 Inclusion and exclusion criteria

Eligible participants were full-time undergraduate students aged ≥18 years who scored ≤13 on the Revised Life Orientation Test (LOT-R), had no significant visual or hearing impairments, reported no history of severe motion sickness, and had no prior experience with the VR games used in the control condition. Exclusion criteria included inability to provide informed consent, current participation in psychosocial therapy, or current use of antidepressant or antipsychotic medications within the past 28 days, in order to ensure that observed effects could be attributed to the PCBT-based VR intervention rather than to concurrent mental health treatments. A prior history of psychotherapy or psychiatric diagnosis, in the absence of ongoing treatment, did not preclude participation.

### 2.3 Interventions

During all VR sessions, adverse events (e.g., cybersickness, dizziness, nausea) were actively monitored using structured verbal symptom checks and continuous observation. Participants were informed that they could stop the session at any time if they experienced discomfort. No adverse events were reported or observed throughout the study.

#### 2.3.1 Pre-intervention training

Participants who met eligibility criteria first completed a brief pre-intervention training session, during which they sat in a quiet room and engaged in a 5-min VR experience to acclimate to the equipment. Following the familiarization period, participants were asked to remain seated quietly with their eyes open for 5 min to standardize pre-session physiological and attentional states. Participants were then randomly assigned to either the experimental or control group using simple randomization procedures. Each participant completed one 30-min session per week for 6 weeks in a distraction-free private room. VR headsets and controllers were disinfected after every session.

#### 2.3.2 Experimental group (PCBT-based VR intervention)

Each session consisted of guided, immersive VR–based PCBT and mindfulness components adapted from validated protocols in prior research (Modrego-Alarcón et al., 2021; Tarrant et al., 2018). The intervention was delivered using a head-mounted display that provided a fully immersive, three-dimensional virtual environment with visual and auditory input, minimizing external sensory distraction. VR modules were developed by XRHealth Inc. and included structured therapeutic content such as body scanning, attentional focus, emotional regulation, relaxed breathing, and progressive muscle relaxation. Participants engaged with the virtual environment through guided narration and visual cues embedded within the scene. Interaction was primarily passive-to-semi-active, involving attentional engagement, guided responses to prompts, and limited controller- or gaze-based input, rather than avatar-based social interaction or open-ended exploration. Different modules were administered across weekly sessions to provide varied but thematically consistent therapeutic experiences.

#### 2.3.3 Control group (task-based VR intervention)

Control group participants received identical VR hardware and engaged in a non-therapeutic, task-based VR game developed by Dr. Shen et al. (2022) that required no prior skills. The control condition was designed to control for VR exposure and engagement without incorporating therapeutic or emotion-focused content, and sessions matched the experimental group in duration and frequency. Using the hand controller, participants cast spells (e.g., bees, bouncy balls, sparkler spells) at virtual objects that reacted differently to each spell, providing an engaging, game-like interaction loop without mindfulness, cognitive restructuring, or emotion regulation components. This design allowed control for immersion, novelty, and cognitive engagement while isolating the therapeutic content delivered through the VR environment.

### 2.4 Sample size

*A priori* power analysis was conducted using G*Power 3.1. Assuming a medium effect size (r = 0.30) between optimism and mental health variables (Lai et al., 2024), *α* = 0.05, and 80% power, at least 84 students were required for screening. For the intervention phase, a repeated-measures ANOVA with an effect size of *f* = 0.30 required 24 participants (12 per group). For four repeated assessments, 18 participants (9 per group) were sufficient to detect within–between interactions.

### 2.5 Measurements

#### 2.5.1 Sociodemographic

Participants were asked to provide their age, gender, race or ethnicity, academic majors, grade levels, and any histories of psychotherapy, psychiatric medication, and psychiatric disorders.

#### 2.5.2 Optimism

Optimism was measured using the 10-item LOT-R, rated on a 5-point Likert scale (Scheier et al., 1994; Przybyszowski et al., 2022). Total scores range from 0 to 24, with higher scores indicating greater optimism. The LOT-R shows good psychometric properties in college students (Cronbach’s *α* = 0.79) (Lai et al., 2024) and in the stroke population (Cronbach’s *α* = 0.82) (Shifren and Anzaldi, 2018), along with test–retest reliability over 4 months in college students (*r* = 0.79) (Scheier et al., 1994).

#### 2.5.3 Stress

Stress was measured using the Perceived Stress Scale (PSS), a 10-item measure assessing stress frequency over the past month using a 5-point Likert scale (Cohen et al., 1983). Scores range from 0 to 40, with higher scores indicating greater perceived stress. Reliability among undergraduates is high (*α* ≈ 0.81) (Lai et al., 2024; Lin et al., 2020).

#### 2.5.4 Anxiety

Anxiety symptoms were assessed using the 7-item Generalized Anxiety Disorder Scale (GAD-7), rated from 0 (‘not at all’) to 3 (‘nearly every day’). Total scores range from 0 to 21 (Spitzer et al., 1960). The GAD-7 shows strong internal consistency in the general population (Cronbach’s *α* = 0.81) (Löwe et al., 2008) and among undergraduate students (Cronbach’s *α* = 0.92) (Lai et al., 2024).

#### 2.5.5 Depression

Depression was measured using the 9-item Patient Health Questionnaire (PHQ-9), rated from 0 to 3. Scores range from 0 to 27, with higher values indicating greater depressive symptoms (Kroenke et al., 2001). The PHQ-9 demonstrates good internal consistency in clinical patients (Cronbach’s *α* = 0.81–0.84) (Kroenke et al., 2016) and excellent internal consistency among college students, with a Cronbach’s *α* of 0.90 (Lai et al., 2024).

### 2.6 Data collection

Baseline sociodemographic information and psychological assessments were collected using Qualtrics surveys at four time points: pre-intervention (T1), post-intervention (T2), 3-month follow-up (T3), and 6-month follow-up (T4). All participants provided informed consent electronically before beginning the survey. Trained assessors blinded to group allocation conducted the outcome assessments. Each participant received a unique identifier to maintain confidentiality and link longitudinal data. All data were stored on secure, password-protected university servers with restricted access.

### 2.7 Data analysis

All analyses were conducted using SPSS 28.0 (IBM Corp.) and the R package *lme4* for mixed-effects modeling (Douglas et al., 2015). Descriptive statistics (means, standard deviations, and frequencies) were used to summarize demographic characteristics and baseline psychological measures. Group differences at baseline were examined using Mann–Whitney *U* tests for continuous variables and chi-square tests for categorical variables.

Longitudinal intervention effects were examined using linear mixed-effects models that incorporated all repeated measurements (T1, T2, T3, and T4). Mixed-effects modeling was selected to maximize statistical efficiency and retain participants with partial follow-up data. In this model structure, the main effect of the intervention (coded as 0 for all control group observations and the experimental group at T1 (baseline); 1 for the experimental group T2, T3, and T4) estimates the immediate level change from baseline to post-intervention. The month variable and its interaction with the intervention group then capture the subsequent longitudinal trajectories (slopes) during the follow-up period. This approach allows for a clear differentiation between the instantaneous treatment effect and the rate of change over time. To distinguish the immediate impact of the intervention from the long-term maintenance of effects, time was treated as a numerical variable representing months since the intervention’s completion. Specifically, baseline (T1) and immediate post-intervention (T2) were both coded as month = 0, while follow-up assessments at 3 months (T3) and 6 months (T4) were coded as month = 3 and month = 6, respectively.

The model included intervention group (PCBT-based VR vs. control), time, and the interaction (group × time) as fixed effects. The interaction tested whether longitudinal change over time differed between groups. Age, gender, and academic period (semester vs. non-semester) were included as covariates. A random intercept for each participant accounted for within-subject correlations due to repeated measures. Model coefficients (*β*), standard errors (SE), and *p*-values were reported for all psychological outcomes, including optimism, stress, anxiety, and depression. Statistical significance was set at *p* < 0.05, with trend-level effects defined as 0.05 ≤ *p* < 0.10. Given the exploratory nature of this pilot study, no correction for multiple comparisons was applied.

## 3 Results

### 3.1 Participant completion rates

**Figure 2:**
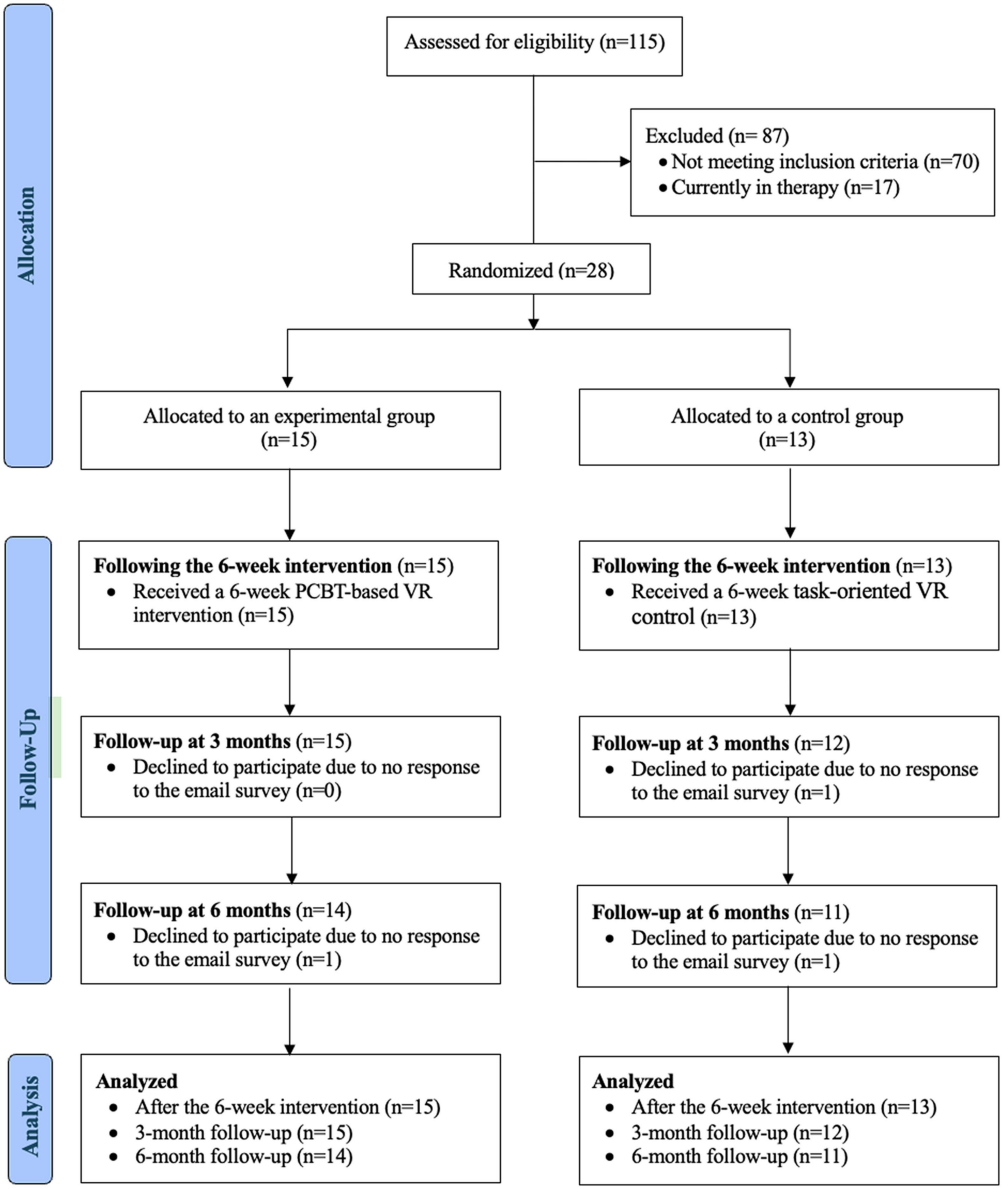
Participant flow diagram for the randomized controlled trial. This diagram shows the number of students invited, enrolled, randomized, and assessed at each time point (T1–T4). Twenty-eight participants were randomized, all completed T1 and T2, 27 completed T3, and 25 completed T4.

Twenty-eight of the 115 invited students enrolled in the study, yielding a recruitment rate of 24.35%. All participants (100%) completed the pre-intervention (T1) and post-intervention (T2) assessments. At the 3-month follow-up (T3), 27 participants (96.43%) completed the assessment, and 25 participants (89.29%) completed the 6-month follow-up (T4) (Figure 2).

### 3.2 Participant characteristics

A total of 28 students participated in the study, with 13 assigned to the control group and 15 to the experimental group (Table 1). The mean age was 20.54 years (SD = 1.39) in the control group and 19.33 years (SD = 1.35) in the experimental group. Most participants identified as female (69.23% in the control group; 60.00% in the experimental group). No significant group differences emerged for gender (*p* = 0.61). The experimental group was slightly younger than the control group (*p* = 0.03); all models were adjusted for age. Racial/ethnic distributions were similar across groups, with African American, Afro-Caribbean, and Non-Hispanic White students comprising the largest categories. No significant between-group differences were observed (*p* = 0.72).

**Table 1:** Baseline sociodemographic characteristics and optimism scores (LOT-R) for the intervention and control groups (*n* = 28).

Variable	Control group (*n* = 13)	Experimental group (*n* = 15)	*U*/*X*^2^	*p*-value
Sociodemographic data				
Age (year, mean ± SD)	20.54 ± 1.39	19.33 ± 1.35	50.50	0.03^a^
Gender, *n* (%)				
Male	4 (30.77)	6 (40.00)	0.26	0.61^b^
Female	9 (69.23)	9 (60.00)		
Race/ethnicity, *n* (%)				
African American, Afro-Caribbean	4 (30.77)	5 (33.33)	2.09	0.72^b^
Asian American	2 (15.38)	4 (26.67)		
Latino or Hispanic American	2 (15.38)	1 (6.67)		
Non-Hispanic White or European American	4 (30.77)	5 (33.33)		
Others	1 (7.69)	0 (0.00)		
Study major, *n* (%)				
Business	0 (0.00)	1 (6.67)	1.29	0.86^b^
Engineering	3 (23.08)	3 (20.00)		
Fine arts, Humanities and Social Sciences	3 (23.08)	4 (26.67)		
Health sciences	4 (30.76)	3 (20.00)		
Sciences	3 (23.08)	4 (26.67)		
Grade levels, *n* (%)				
Freshmen	2 (15.38)	7 (46.67)	4.39	0.22^b^
Sophomore	2 (15.38)	1 (6.67)		
Junior	4 (30.77)	5 (33.33)		
Senior	5 (38.46)	2 (13.33)		
History of psychotherapy, *n* (%)				
No	13 (100)	12 (80.00)	2.91	0.23^b^
Yes	0 (0.00)	2 (13.33)		
Unsure	0 (0.00)	1 (6.67)		
History of psychiatric medication, *n* (%)				
No	13 (100)	14 (93.33)	0.89	0.34^b^
Yes	0 (0.00)	0 (0.00)		
Unsure	0 (0.00)	1 (6.67)		
History of psychiatric disorder, *n* (%)				
No	9 (69.23)	11 (73.33)	0.14	0.93^b^
Yes	2 (15.38)	2 (13.33)		
Unsure	2 (15.38)	2 (13.33)		
LOT-R, mean ± SD	10.08 ± 2.63	10.20 ± 2.51	94.50	0.89^a^

Continuous variables were compared using the Mann–Whitney *U* test,^a^ and categorical variables were compared using the chi-square (*χ*^2^) test.^b^ Values are presented as mean ± SD for continuous variables and *n* (%) for categorical variables. SD, standard deviation; LOT-R, Life Orientation Test–Revised. Reported histories of psychotherapy, psychiatric medication use, or psychiatric disorders reflect prior treatment or diagnosis; participants were not receiving psychosocial therapy or psychotropic medication at the time of enrollment.

Participants represented diverse academic majors and class years, with no significant differences between groups in either distribution (both *p* = 0.86). Most students reported no prior psychotherapy, psychiatric medication use, or psychiatric diagnoses, with no significant differences between groups (all *p* > 0.23). Participants who reported a history of psychotherapy, psychiatric medication use, or psychiatric diagnosis were not receiving active treatment at the time of enrollment and therefore met the study’s eligibility criteria. Baseline optimism scores did not differ significantly between groups (*p* = 0.89).

### 3.3 The short-term effect of the PCBT-based VR intervention

Short-term intervention effects were evaluated using the main effect of intervention in the mixed-effects model, reflecting group differences observed during the early post-intervention period. The model indicated a significant short-term increase in optimism among participants in the PCBT-based VR group (*β* = 2.02, SE = 0.77, *p* = 0.01) and a significant reduction in depressive symptoms (*β* = −3.04, SE = 1.15, *p* = 0.01). Anxiety showed a trend toward short-term reduction (*β* = −1.55, SE = 0.84, *p* = 0.07), although this effect did not reach statistical significance, and stress levels did not significantly change during this period (*β* = −0.75, SE = 1.27, *p* = 0.56) (Table 2). Collectively, these findings suggest that the PCBT-based VR intervention produced notable early improvements in optimism and depression, with smaller or nonsignificant short-term effects on anxiety and stress.

**Table 2:** Short-term intervention effects during the early post-intervention period (*n* = 28).

Outcome	Beta (*β*)	SE	*p*-value
LOT-R	2.02	0.77	0.01
PSS	−0.75	1.27	0.56
GAD-7	−1.55	0.84	0.07
PHQ-9	−3.04	1.15	0.01

Mixed-effects model estimates reflect the main effect of intervention, representing early post-intervention group differences between the PCBT-based VR and control groups. Positive *β* indicates an increase and negative *β* a decrease in the outcome. Estimates are adjusted for age, gender, and academic period. *p* < 0.05 indicates significance; 0.05 ≤ *p* < 0.10 indicates a trend. SE, standard error; LOT-R, Life Orientation Test–Revised; PSS, Perceived Stress Scale; GAD-7, Generalized Anxiety Disorder-7; PHQ-9, Patient Health Questionnaire-9.

### 3.4 The long-term effect of the PCBT-based VR intervention

Long-term effects were examined using the mixed-effects model by assessing the month × intervention interaction term. As shown in Table 3, the month × intervention interaction was not significant (*β* = −0.32, SE = 0.20, *p* = 0.12), indicating that longitudinal change in optimism did not significantly differ between groups. Optimism scores increased over time for participants in both groups (*β* = 0.38, SE = 0.16, *p* = 0.02). Consistent with this pattern, interaction effects were also nonsignificant for anxiety (*β* = 0.33, SE = 0.21, *p* = 0.13), depression (*β* = 0.51, SE = 0.30, *p* = 0.09), and stress (*β* = 0.53, SE = 0.34, *p* = 0.13) (Table 4). Taken together, these findings indicate that the PCBT-based VR intervention was associated with early post-intervention improvements in optimism. While these initial gains were maintained across the follow-up period, the long-term trajectories did not significantly differ between the intervention and control groups.

**Table 3:** Mixed-effects model estimates for long-term effects on optimism (LOT-R).

Covariates	Beta (*β*)	SE	*p*-value
Intervention	2.02	0.77	0.01
Months after intervention	0.38	0.16	0.02
Intervention × months	−0.32	0.20	0.12
Age	0.26	0.33	0.44
Gender	−0.24	0.97	0.80
Academic period	0.31	0.70	0.66

Mixed-effects model estimates for the long-term analysis of optimism, including the main effects of the intervention, time in months since baseline, and their interaction, along with covariates (age, gender, and academic period). Positive *β* values indicate increases in optimism, and negative *β* values indicate decreases. SE, standard error.

**Table 4:** Mixed-effects model interaction estimates (month × intervention) for psychological outcomes.

Outcome	Beta (*β*)	SE	*p*-value
LOT-R	−0.32	0.20	0.12
PSS	0.53	0.34	0.12
GAD-7	0.33	0.21	0.13
PHQ-9	0.51	0.30	0.09

This table presents the month × intervention interaction terms from the mixed-effects models for each psychological outcome, evaluating whether longitudinal change differed between the PCBT-based VR intervention and control groups. Positive *β* values indicate a greater increase (or slower decrease) in the outcome over time in the intervention group relative to the control group, while negative *β* values indicate the opposite pattern. SE, standard error; LOT-R, Life Orientation Test–Revised; PSS, Perceived Stress Scale; GAD-7, Generalized Anxiety Disorder-7; PHQ-9, Patient Health Questionnaire-9.

## 4 Discussion

This study examined the short-term and long-term effects of a PCBT-based VR intervention on optimism and mental health in college students, compared to a task-oriented VR control condition. Results indicated that the PCBT-based VR intervention produced significant short-term improvements in optimism and depressive symptoms among students with low optimism, with these optimism gains lasting for 6 months. However, there were no significant long-term differences in optimism, stress, anxiety, or depression between the groups, implying that ongoing reinforcement might be necessary to achieve or maintain additional long-term benefits.

### 4.1 Effects on optimism

This study showed that the PCBT-based VR intervention produced a substantial early advantage in optimism relative to the control condition, and this difference was maintained over time. Although both groups improved at a similar rate during follow-up, the experimental group maintained a higher level of optimism throughout the observation period, suggesting the potential of structured, technology-based interventions to enhance optimism among college students. Although some prior research suggested that PCBT interventions may not yield statistically significant changes in optimism among patients with major depressive disorder, they still showed higher mean optimism scores compared to baseline (Geschwind et al., 2019). This suggests that while PCBT-based VR approaches may not fully eliminate pessimistic thinking in clinical populations, they can promote meaningful gains in optimism, which is a known protective factor for mental health and may reduce vulnerability to stress and depressive symptoms in non-clinical student samples. This finding suggests that optimism increased over time in both groups, supporting the observation that participants’ optimism scores rose gradually across months following the intervention. Similarly, Geschwind et al. (2019) reported improvements in optimism scores after treatment, with participants reaching levels comparable to normative population averages. However, the negative intervention-by-time interaction was not statistically significant (*p* = 0.12), indicating that long-term optimism trajectories did not differ significantly between groups over the follow-up period. This pattern reflects broader evidence indicating that, without reinforcement or booster sessions, the effects of psychological interventions often attenuate. Consistent with this, meta-analyses of positive psychology interventions have found that initial gains in optimism tend to diminish unless interventions are maintained or repeated (Bolier et al., 2013). The PCBT-based VR intervention produced a significant short-term improvement in optimism among students with low baseline optimism, consistent with prior work showing that PPIs and PCBT can enhance positive expectations and cognitive appraisal (Bolier et al., 2013; Sin and Lyubomirsky, 2009; Lyubomirsky and Layous, 2013). VR immersion may further support these effects by enhancing attentional focus and emotional engagement (Li Pira et al., 2023; Xu et al., 2024; Tarrant et al., 2018). Although optimism remained higher in the intervention group through follow-up, the nonsignificant month × intervention interaction indicates that the rate of long-term change was similar between groups. This suggests that early gains were sustained but did not continue to increase in the absence of reinforcement (Geschwind et al., 2019; Lyubomirsky and Layous, 2013).

### 4.2 Effects on mental health outcomes

After the intervention, the experimental group experienced significant reductions in depression and trend-level reductions in anxiety compared to the control group in the short term; however, the difference was not statistically significant. The finding was aligned with previous research showing that PCBT interventions can effectively reduce depressive symptoms in patients with major depressive disorder (Xu et al., 2024; Freeman et al., 2017; Geschwind et al., 2019). Additionally, online PPIs have been shown to decrease depressive symptoms among Chinese college students (Auyeung and Mo, 2019). University students who participated in PPIs also reported significantly lower levels of depression (Tarrats-Pons et al., 2025). Moreover, some meta-analysis studies further support these findings, indicating that PPIs reliably lead to significant reductions in depression among adults aged 18 and older (Bolier et al., 2013; Sin and Lyubomirsky, 2009; Lim and Tierney, 2023). Furthermore, the decrease in anxiety scores among undergraduate students after this intervention aligns with existing studies that have shown that an interactive virtual environment and VR exposure therapies can reduce anxiety in clinical patients (Freeman et al., 2017; Felnhofer et al., 2019). These findings suggest that combining PCBT principles with immersive VR delivery offers a promising approach for addressing depressive and anxiety symptoms in university settings by integrating evidence-based cognitive restructuring techniques with the engagement and accessibility of technology-based interventions.

The intervention also produced significant short-term reductions in depressive symptoms and a trend toward reduced anxiety, aligning with evidence that PCBT and VR-based approaches can improve emotional regulation and reduce negative affect (Xu et al., 2024; Freeman et al., 2017; Geschwind et al., 2019). These short-term benefits mirror findings from other PPIs implemented among university students (Bolier et al., 2013; Sin and Lyubomirsky, 2009; Auyeung and Mo, 2019). However, consistent with the optimism results, long-term slopes did not differ between groups, suggesting that the intervention’s mental health effects were strongest during the active treatment phase and did not diverge from the control condition over time.

### 4.3 Interpretation of short-term and long-term patterns

Taken together, the results indicate that the PCBT-based VR intervention is most effective for producing short-term cognitive and emotional benefits, which were maintained but not amplified across 6 months. This pattern is consistent with the broader PPI and PCBT literature, which often reports strong immediate gains that level off without continued practice or booster sessions (Geschwind et al., 2019; Lyubomirsky and Layous, 2013). Additionally, natural academic and environmental fluctuations common in college populations may influence long-term psychological trajectories (American College Health Association, 2024), underscoring the value of incorporating ongoing support to sustain intervention effects.

### 4.4 Implications for VR-delivered positive psychological interventions

The findings suggest that VR-delivered PCBT may be a practical and engaging approach for supporting psychological wellbeing in college students. The immersive nature of VR can enhance user engagement and attentional focus, offering a structured environment for practicing positive cognitive and emotional skills (Xu et al., 2024; Freeman et al., 2017; Geschwind et al., 2019). These features may be especially useful for students who prefer technology-based mental health resources or who face barriers to participating in traditional services. The intervention’s ability to produce early improvements that were maintained across several months also highlights its potential as a scalable, low-stigma tool for strengthening positive cognitive resources such as optimism. However, because long-term trajectories did not differ between groups, future VR-based PPIs may benefit from incorporating booster sessions or continued practice opportunities to help sustain and strengthen initial gains.

### 4.5 Limitations

Several limitations should be considered when interpreting these findings. First, the study was conducted with a small sample from a single public university, which may limit the generalizability of the results. Second, all outcomes were assessed using self-report measures, which may be influenced by social desirability or limited self-awareness (FvdM, 2008; Paulhus and Vazire, 2007). Third, although participants were randomly assigned, the groups differed in age at baseline, which could have influenced outcomes despite statistical adjustment. The significant baseline age difference between groups represents an additional limitation. Participants in the intervention group were, on average, younger than those in the control group, which may have influenced engagement with the VR technology. Younger participants may be more accustomed to immersive digital environments and interactive technologies, potentially leading to greater comfort, immersion, or responsiveness to VR-based interventions. Although age was included as a covariate in the analyses, the small sample size limits conclusions regarding age-related effects. Future studies should aim to more closely match groups on age or examine age as a potential moderator of engagement and intervention efficacy.

Fourth, the intervention period coincided with typical academic fluctuations, which may naturally affect stress, mood, and optimism among undergraduate students (American College Health Association, 2024; Lin et al., 2020). In addition, the VR-based intervention was conceptualized and implemented as a research-oriented, wellness-focused intervention rather than a clinically regulated therapeutic product intended for diagnostic or treatment decision-making. Formal regulatory classification, such as consideration under Software as a Medical Device (SaMD) frameworks, was beyond the scope of this pilot study and was not addressed. This limits conclusions regarding its clinical deployment, regulatory pathway, and scalability beyond research or wellness settings.

Although attrition was low, any loss to follow-up may introduce bias if participants who withdrew differed from those who completed all assessments. Finally, although students with severe psychiatric conditions were excluded, a small number of participants reported a prior history of psychotherapy or psychiatric treatment. While this approach enhanced ecological validity, it may limit the generalizability of the findings and influence the interpretation of intervention effects. Future studies with larger samples should apply stricter inclusion criteria or conduct subgroup analyses to better examine whether prior treatment history moderates intervention outcomes. Taken together, these limitations highlight the need for larger, multisite studies that incorporate objective measures, evaluate regulatory considerations, and further clarify the clinical and translational positioning of VR-based mental health interventions.

### 4.6 Future directions

Future research should build on this pilot study by recruiting larger and more diverse samples across multiple university settings to enhance generalizability. Expanding the intervention to include booster sessions or sustained practice components may help strengthen and maintain long-term effects, consistent with evidence that ongoing engagement is often necessary to preserve gains in positive psychology and CBT-based programs (Geschwind et al., 2019; Lyubomirsky and Layous, 2013). Incorporating qualitative methods, such as interviews or open-ended feedback, could provide deeper insight into participants’ experiences, perceived benefits, and barriers to engagement. Additionally, examining objective indicators—such as physiological stress markers or behavioral measures—may complement self-reported data and reduce the influence of reporting biases (FvdM, 2008; Paulhus and Vazire, 2007). Finally, comparing VR-delivered PCBT with alternative digital or in-person PPIs may help clarify which delivery formats are most effective, accessible, and feasible for college populations.

## 5 Conclusion

In conclusion, this study provides preliminary evidence that a PCBT-based VR intervention can produce meaningful short-term improvements in optimism and depressive symptoms among college students with low baseline optimism. These early gains were maintained over the 6-month follow-up period; however, long-term trajectories of optimism, stress, anxiety, and depression did not differ significantly between the intervention and active control groups. This pattern suggests that while VR-delivered PCBT may support short-term enhancement and maintenance of positive cognitive resources, it did not demonstrate a superior long-term effect relative to an active VR control. Continued practice or booster support may be necessary to achieve additional or sustained improvements beyond the initial intervention period. As VR technologies become increasingly accessible, VR-delivered PCBT may serve as an engaging, scalable, and low-stigma approach to supporting student wellbeing. Larger, multi-site trials are needed to confirm these findings and further examine strategies to enhance long-term effectiveness.

## Data Availability

The raw data supporting the conclusions of this article will be made available by the authors, without undue reservation.
